# The multi-dimensional nature of vocal learning

**DOI:** 10.1098/rstb.2020.0236

**Published:** 2021-10-25

**Authors:** Sonja C. Vernes, Buddhamas Pralle Kriengwatana, Veronika C. Beeck, Julia Fischer, Peter L. Tyack, Carel ten Cate, Vincent M. Janik

**Affiliations:** ^1^ School of Biology, University of St Andrews, St Andrews, UK; ^2^ Neurogenetics of Vocal Communication Group, Max Planck Institute for Psycholinguistics, Nijmegen, The Netherlands; ^3^ Donders Institute for Brain, Cognition and Behaviour, Nijmegen, The Netherlands; ^4^ Institute of Biodiversity, Animal Health and Comparative Medicine, University of Glasgow, Glasgow, UK; ^5^ Department of Behavioural and Cognitive Biology, University of Vienna, Vienna, Austria; ^6^ Cognitive Ethology Laboratory, German Primate Centre, Göttingen, Germany; ^7^ Department of Primate Cognition, Georg-August-University Göttingen, Göttingen, Germany; ^8^ Institute of Biology, Leiden University, Leiden, The Netherlands

**Keywords:** vocal learning, songbird, cognition, behaviour, evolution, language

## Abstract

How learning affects vocalizations is a key question in the study of animal communication and human language. Parallel efforts in birds and humans have taught us much about how vocal learning works on a behavioural and neurobiological level. Subsequent efforts have revealed a variety of cases among mammals in which experience also has a major influence on vocal repertoires. Janik and Slater (*Anim. Behav.* **60**, 1–11. (doi:10.1006/anbe.2000.1410)) introduced the distinction between vocal usage and production learning, providing a general framework to categorize how different types of learning influence vocalizations. This idea was built on by Petkov and Jarvis (*Front. Evol. Neurosci.* **4**, 12. (doi:10.3389/fnevo.2012.00012)) to emphasize a more continuous distribution between limited and more complex vocal production learners. Yet, with more studies providing empirical data, the limits of the initial frameworks become apparent. We build on these frameworks to refine the categorization of vocal learning in light of advances made since their publication and widespread agreement that vocal learning is not a binary trait. We propose a novel classification system, based on the definitions by Janik and Slater, that deconstructs vocal learning into key dimensions to aid in understanding the mechanisms involved in this complex behaviour. We consider how vocalizations can change without learning, and a usage learning framework that considers context specificity and timing. We identify dimensions of vocal production learning, including the copying of auditory models (convergence/divergence on model sounds, accuracy of copying), the degree of change (type and breadth of learning) and timing (when learning takes place, the length of time it takes and how long it is retained). We consider grey areas of classification and current mechanistic understanding of these behaviours. Our framework identifies research needs and will help to inform neurobiological and evolutionary studies endeavouring to uncover the multi-dimensional nature of vocal learning.

This article is part of the theme issue ‘Vocal learning in animals and humans’.

## 1. Introduction

Interest in vocal learning skills has been with us from the beginning of animal behaviour research, with scholars as early as Darwin [1] recognizing the role of learning in the development of bird song and its parallels with human vocal performance. Janik & Slater [2,3] introduced a framework that distinguished between vocal usage learning, in which existing signals are given in a new context or sequence, and vocal production learning, in which signals are modified in form after experience with the signals of others. Petkov & Jarvis [4] noted the continuous nature of this trait, and others have further explored these ideas (e.g. [5–10]). Yet, with more studies providing empirical data, the limits of the initial frameworks become apparent. We build on the definitions proposed by Janik & Slater [2] to refine the categorization of vocal learning in light of the advances made since its publication. Our motivation is that, despite the advances made in the last 20 years and more of research since the first publication of this framework, identifying which species are vocal learners still remains a challenging task. Outside the few highly studied and easily recognizable vocal production learners, membership of this select club is still hotly debated. Furthermore, despite most researchers agreeing that vocal learning is not a binary trait, a satisfactory typology of its components is still lacking.

Vocalizations may vary for a range of reasons, but vocal learning crucially requires a learning process to be the driver of such variation. Although many definitions of learning exist (e.g. see [11]), we have adopted the definition of learning as a modification of an individual's behaviour owing to information memorized from previous experience [12]. Vocal production learning was defined by Janik & Slater [2] as the production of modified or novel vocalizations, as a result of learning from the experience of the acoustic signals of others. Central to this definition is that auditory input from a model sound leads to the formation of a memory of the sound (a template), to which the vocal output is compared. We maintain this definition and highlight that the auditory model may be a range of sounds including vocalizations produced by another animal (recorded or live), synthesized playbacks or even non-vocal mechanical sounds (e.g. lyrebirds' imitation of a chainsaw or camera shutter).

We classify behavioural changes associated with vocal learning into a cluster of discrete dimensions and then consider how these behaviours relate to evidence for or against underlying mechanisms. This approach explicitly acknowledges that there may not be a single vocal learning phenotype, rather multiple components, expressed to different degrees. We first consider cases where changes in vocalizations may be occurring that are not the result of learning, to distinguish them from learned modifications (§2). We then explore the definitions of, and distinctions between, vocal usage learning and vocal production learning. Usage learning is a form of contextual learning in which a signal is produced in a new context as a result of experience [2]. This term covers a range of behaviours where the context may refer to environmental or behavioural contexts (natural or artificial), and can include positional contexts (i.e. position in a sequence of calls). For usage learning (§3), we consider two dimensions: how vocalization context (behavioural contexts and positional context) or timing (call timing and rhythmicity) is learned. For vocal production learning itself (§4), we describe three dimensions: the copying of auditory models (convergence or divergence on model sounds, accuracy of copying), the degree of change (type of vocal modification and breadth of learning) and timing (when learning takes place, the length of time it takes and how long it is retained). We also consider the grey areas that currently hinder clear classification (§5), our current mechanistic understanding of these traits (§6) and outstanding questions for future research (§7 and Box 1).

Box 1. **Key outstanding questions**

**Behaviour**

— During which life stages are animals capable of vocal learning and how (if at all) do the mechanisms that enable vocal production learning early in life differ from those that allow production learning later in life?
— How widespread is learning to use calls in a new sequence?
— Can the production of specific vocal rhythms be learned by animals?

**Mechanisms**

— What are the mechanistic underpinnings of the dimensions of vocal production learning?
— What are the mechanisms underlying the different phenomena described as usage learning and how do they relate to vocal production learning mechanisms?
— To what extent do usage learning and production learning share neural mechanisms?
— What are the mechanisms underlying sequence learning, and how do they relate to usage or production learning mechanisms?
— Is the process of modifying existing vocalizations mechanistically continuous or distinct from that of producing novel vocalizations?
— To what extent are auditory model guided and reward-based vocal modifications based on the same or different neural mechanisms?
— What neural and physiological constraints determine the vocal learning abilities of each species?
— How do neural control mechanisms of vocal production learning in laryngeal and non-laryngeal sound production compare?

**Evolution**

— How widespread are different types of vocal learning and how conserved over evolution are the mechanisms and circuits driving vocal learning in birds and mammals?
— What are the contexts in which (various types of) vocal learning may have arisen (e.g. socioecological factors)
— What are the selection processes (e.g. group member or individual recognition, kin selection, inter- and intra-sexual selection) that shaped vocal learning abilities in different species?

Our framework gives us a means to directly compare the different aspects of vocal learning abilities across animals. In this way, we aim to make clear the research directions that are needed to close current gaps in knowledge and make significant strides in understanding the remarkable trait of vocal learning. We hope this framework will make it possible to investigate and reveal the mechanisms that drive each dimension, both in individual species, or in a true ‘like-with-like’ cross-species approach, and in this way, better understand the prevalence and evolution of this complex phenomenon.

## 2. Non-learned inputs into vocal variation

To make headway in the identification of vocal learning, it is necessary to first recognize other factors that may contribute to variation, but that do not involve the learned adjustment of vocal output.

### (a) Variation in response to the acoustic environment

Animals modify the intensity, frequency, duration and repetition of their signals to compensate for variation in acoustic conditions in their environment (see [13–16]). If these adjustments involve immediate audio-vocal feedback [17–19] rather than memorized information from previous experience, they would not be classed as learned changes, based on the definition of learning applied herein. This includes phenomena such as the jamming avoidance response of some bat species [20].

Highly sophisticated or precise vocal change in response to auditory input does not necessarily indicate that the change is learned. One sophisticated phenomenon that involves auditory–vocal feedback but is not generally defined as vocal learning is the Doppler shift compensation (DSC) of Rhinolophid bats. When a bat hears a Doppler-shifted echo, it precisely modifies the frequency of its call so that the echo stays in a preferred frequency band [21]. DSC is achieved by auditory–vocal feedback mechanisms in the midbrain that do not require learning under our definition [22]. Thus, to classify observed vocal variation in response to environmental sounds, it is critical to determine if the change is a non-learned response (such as DSC), or whether the animal employs a learning process to modify its vocal output.

### (b) Controlling for other sources of acoustic variation

Changes owing to physical growth and maturation (e.g. [23]), rather than changes in auditory experience, can cause a shift in vocal characteristics (e.g. fallow deer (*Dama dama*), [24]). Changes in arousal, stress, disease and hormonal or emotional state (e.g. [25,26]) can also account for changes in acoustic structure [27]. For example, the rate of parental vocal input was shown to positively affect the rate of vocal maturation in common marmoset young [28,29]. These differences may be explained by an increased effort in practising driving an existing developmental programme more rapidly. Stress, e.g. induced by separation, can have a direct impact on the spectral structure of calls in common marmosets [30] and the rate of parental vocal input can have a stress alleviating effect. Acoustic features of calls may also be influenced by the reproductive state of the subjects [31]. Therefore, the contribution of physiological and morphological factors to changes in vocalizations should be considered before inferring vocal learning.

## 3. Vocal usage learning

Usage learning and vocal production learning are two different facets of how learning can affect vocal behaviour. Usage learning can be applied to existing vocalizations or those acquired by vocal production learning, and some animals are capable of both. So, while usage and production learning can co-occur, usage learning does not itself require vocal production learning. For instance, if a young animal learns to restrict the use of an alarm call to a specific set of predators, but the alarm call itself does not require learning to develop, this is a clear example of usage learning. However, if an animal learns to produce a novel vocalization to signal the presence of predators this would involve vocal production learning (to learn the novel vocalization) and usage learning (to learn the context in which to employ it).

Vocal usage learning appears to be fairly common in mammals and birds. Adret [32] and Janik & Slater [2] highlighted usage learning in a broad group of species including parrots, chickens, budgerigars, mynahs, cats, dogs, rats, lemurs, sea lions and dolphins. More recent studies add bats [33], elephants [34] and seals [35] to this list. Nevertheless, usage learning can take various different forms and levels of complexity and describing these quite diverse phenomena under the label of usage learning does not imply that a single mechanism or the same one underlies them all. For example, primatologists have proposed that non-human primates have a special propensity for usage learning that is a precursor to learning in human language development [9,36]. Below we describe some of the different contexts in which the use of signals can change through usage learning.

### (a) Learning to use calls in a new context

The most widely recognized form of usage learning is learning to produce an existing call in a new environmental context. A clear example of this is a study on European blackbirds (*Turdus* *merula*). Perceiving a mobbing conspecific together with a novel, harmless stuffed bird (a honey eater) induced blackbirds to mob the innocuous object using specific mobbing vocal behaviour (e.g. ‘duck’ calls) normally given to predators. This behaviour could be culturally transmitted along a chain of at least six individuals [37].

A common way of demonstrating learning to use calls in a new context is by training an animal to produce vocalizations in response to a conditioned stimulus [2] such as an arbitrary hand or light signal, which can be considered a fairly simple form of contextual usage learning. Studies on a variety of species used such a method to demonstrate conditioned control over a single call type [32]. More sophisticated control over vocal usage was shown by crows that were able to produce or withhold vocalizing to different colour cues [38]. Rhesus monkeys (*Macaca mulatta*) [39], gray seals (*Halichoerus grypus*) [35] and bottlenose dolphins (*Tursiops truncatus*) [40] were even able to learn to produce more than one call type in response to different arbitrary signals. Note that, in the case of the dolphins, these were novel synthetic calls that were learned and used in an arbitrary context [40], suggesting a case where both vocal production and usage learning were occurring. Another method is to link food delivery to the production of specific signal parameters without training animals to respond to specific signals. Pale spear-nosed bats (*Phyllostomus discolor*) shifted to rewarded versions of their social calls in such a setup [33].

Identifying usage learning in the wild is more challenging. Animals often change how they use calls, as well as which calls they use over the course of their development but as before it is difficult to distinguish changes caused by maturation or stress from those caused by learning. Natural experiments can help here when populations differ in the number of examples provided to a developing animal. For example, groups of vervet monkeys (*Cercopithecus aethiops*) with more intergroup encounters learned the correct use of ‘wrr’ calls more quickly than groups with fewer encounters [41]. Another interesting context in which usage learning might occur is the deceptive use of calls. For example, several species occasionally produce alarm calls in the absence of any predators, often gaining access to food in the process (e.g. [42]). If shown not to be a production error, the development of this behaviour is likely to involve usage learning. Fork-tailed drongos (*Dicrurus adsimilis*) also produce alarm calls of different species depending on what species are in their audience [43]. Drongos use production learning to acquire the alarm calls of other species, and usage learning to learn the context in which they are given. Developmental studies of these behaviours will be able to shed further light on the role of usage learning in such contexts.

Animals may also learn to produce calls in a new positional context or sequence [2,5]. This can involve the sequencing of calls from an existing repertoire or the sequencing of novel calls acquired through vocal production learning. The arrangement of calls can be in simple sequences, or can apply complex syntactic rules. Arranging or rearranging the sequence of calls or song elements might occur in various ways and through various mechanisms. Depending on the process involved in specific cases, such sequencing may be classified as usage learning or as production learning, or may not fit neatly into these categories. For this reason, we address sequence learning in §5 and acknowledge we need a deeper understanding of the mechanisms underlying sequence learning in the future.

### (b) Learning timing of call use: vocal interactions and rhythms

Coordination of call timing is widespread across the animal kingdom, from coordination of utterances in human conversations [44], to temporally entrained advertisement calls of male frogs [45]. Duetting may involve temporal coordination such that vocalizations overlap, or occur in a turn-taking pattern with calls and their responses being produced within a limited time window. The widespread nature of this ability has suggested it as relevant for the evolution of communication in animals and humans [46]. However, turn taking does not necessarily require learning and it is important to make a distinction between those temporal entrainments that are learned, those that are not learned, and those for which learning has not yet been demonstrated.

Temporal coordination of calls often develops very early, making it challenging to determine if the behaviour is learned or not, but examples where call timing influenced by learning is particularly apparent are in the development of duetting displays in birds [47] and mammals [48]. Some bird species produce simple call and response vocalizations, while others produce highly sophisticated duets that are so precisely timed that it can be difficult to tell when one bird stops singing and another continues the song (e.g. [49]). In canebrake wrens (*Cantorchilus zeledoni*), juveniles start duetting by singing together with adults, and their coordination of songs becomes better over time [50]. Furthermore, adults that acquire a new partner have poor coordination directly after pairing, but this improves the longer they duet together [47]. The same has been found in gibbons [48,51]. These findings suggest that canebrake wrens and gibbons learn the appropriate temporal coordination for duetting.

Human and non-human primates (e.g. common marmosets) demonstrate vocal turn-taking behaviour from early in life [28,44,52,53]. Many other mammals such as cetaceans [54], bats [55], elephants [56,57], meerkats [58] and naked mole rats [59] also show turn-taking behaviour, although in most cases learning of timing has not been definitively demonstrated in mammals. By contrast, there are some vertebrates where temporal vocal coordination can clearly be attributed to a central nervous system oscillator that is responsive to call perception, rather than a learned mechanism, such as in rhythmic calls of some anurans [45] or suppression of motor pattern generators in response to hearing conspecific calls in monkeys [60].

Another form of usage learning found in human speech and song is copying novel rhythms in vocalization patterns. Learning to produce novel vocal rhythms has not been demonstrated in animals even though examples of entrainment of motor responses to acoustic rhythms exist [61,62]. Zebra finches (*Taeniopygia guttata*) can create a type of rhythm by predicting the timing of a regular jamming signal and adjusting their own vocalizations to avoid it [63]. They can also learn to distinguish perceptually between a specific isochronous and irregular beat pattern, but do not generalize this to tempo changes of the same patterns, suggesting that they do not have a representation of the global rhythm [64]. Sperm whales (*Physeter macrocephalus*) use rhythmic patterns of clicks, called codas, for communication [65]. Codas are shared across multiple groups, with multiple sympatric vocal clans in the Pacific and Atlantic Oceans [66]. This distribution of codas suggests that whales learn group-distinctive calls through memorizing the rhythmic patterns of their click sounds. Sperm whales are able to precisely time their clicks to match the depth sounder on a ship [67], but learning of rhythmic patterns like those of codas has not been demonstrated. Birds and mammals clearly pay attention to aspects of rhythm in conspecific vocalizations (e.g. [68,69]) but production studies demonstrating vocal learning of rhythm in animals are still missing.

### (c) Constraints and flexibility of usage learning

Training animals to produce their own vocalizations on command is a common way of demonstrating usage learning. As noted above, many species have demonstrated this ability, but few studies have explored how flexible this form of learning is. For example, many species have been trained to produce a signal from their own repertoire on command, but it is unknown if they could be trained to use all of their existing call types in this fashion, or only some of them. The level of difficulty of usage learning in such studies is often reflected in the time required to learn to produce existing calls in response to different signals [70]. In relation to sequence learning, it is also an open question whether animals are able to re-arrange all elements in a sequence arbitrarily or whether there are constraints. There are often predispositions in bird song learning for conspecific song elements and structure [71,72]. It is, therefore, likely that there are limits to how units are recombined since models with unusual syllable combinations may not fit the species template for the conspecific song. More studies are needed to explore such limitations systematically.

A good example of complexity in usage learning is the fine tuning of the contexts in which vervet monkeys give alarm calls during their development [73]. While they seem to be predisposed from birth to produce an aerial alarm to threats in the sky, the attention to detail required to learn the correct predator species for the call moving from broader categories to more specific ones is considerable. This probably relies on different neural processing than that involved in learning to produce an unspecified vocalization on command in a conditioning test. The most detailed documentation of usage learning complexity comes from language studies with grey parrots (*Psittacus erithacus*) that had to harness usage learning to use human words (which were initially acquired by production learning) to refer to objects and give information about their properties [74]. While speech learning in grey parrots is impressive, their usage learning abilities are not unbounded. For example, the acquisition of multiple labels for the same object is much more difficult than the learning of the initial label [75]. All of these examples are case studies in which usage learning was part of a larger investigation of cognitive skills.

To conclude, it is clear that the term ‘usage learning’ covers a broad category of phenomena, most likely involving a range of different mechanisms. A more systematic investigation of usage learning, its diversity, complexity and its constraints would be of great value.

## 4. Vocal production learning

The classical view of vocal production learning is based on studies of humans and songbirds. In this process, the animal uses auditory input from a model sound to produce copies of various degrees of fidelity, depending on the species. Producing a copy of an auditory model is also known from other animal groups, such as parrots [76], pinnipeds [77] and dolphins [40]. These represent clear cases of vocal production learning, where it can be directly shown that individuals exposed to different model sounds start selectively producing what they have heard. In most songbirds, the neural representation of the model is built up from several exposures and stored as the ‘template’ on which later vocal production is modelled. However, there are also examples in which the copying of a model is almost instantaneous, suggestive of an ability for real-time vocal matching. It is not always clear whether this results in a longer lasting or a perhaps more transient neural representation of the auditory model.

Our definition of vocal production learning requires auditory input. However, it is important to note that this does not imply that auditory cues are the only factors involved in this learning process. It has been clearly shown that other factors, such as social interactions, can influence how effectively animals learn during vocal production learning. In humans and songbirds, learning is more effective when interaction with a live tutor is possible, compared to auditory only or even audio-visual playback on a screen [78–80]. As such our definition denotes only the requirement for exposure to an auditory model to form a template during vocal production learning, and should not be interpreted as an exclusion of other contextual factors that clearly play an important role in this process.

**Figure 1.**
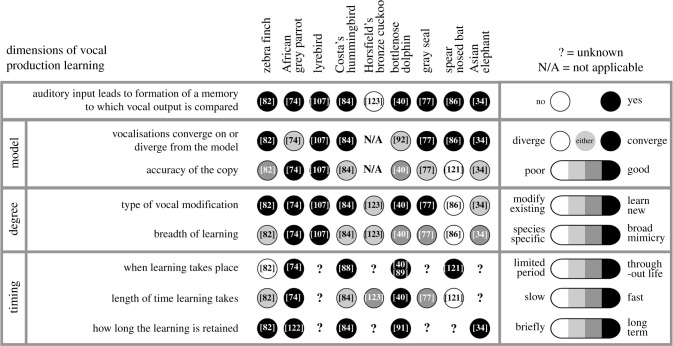
The multi-dimensional nature of vocal production learning. Vocal production learning is the production of modified or novel vocalizations, as a result of learning from the experience of the acoustic signals of others. In this process, auditory input leads to the formation of a memory, to which the vocal output is compared. Examples of avian and mammalian vocal production learning species are represented across the dimensions of vocal production learning outlined in §4. The Horsfield's bronze cuckoo is also included based on our discussion of its inclusion in the wider definition of vocal production learning (see §5). This is not an exhaustive list, rather a selection of species with shared and contrasting abilities. Categorizations are made for each species using discrete or continuous scales, as indicated in the legend. This schema can be used to take an in depth look at the vocal learning properties of an individual species (by looking down a column) or to compare abilities within a single dimension of vocal learning (by looking along a row). Categorizations are intended to indicate what each species has been observed to be capable of doing, and may not reflect their behaviour in their normal environment, or the full extent of their capacity. For example, Asian elephants have been demonstrated to have a high breadth of learning largely based on an Asian elephant in human care that learned human speech-like sounds [34]. However, this behaviour is not observed in normally reared Asian elephants in the wild. African elephants in human care are reported to imitate the sounds of Asian elephants and of trucks [81], but the breadth of mimicry for copying other sounds in elephants is not known. A question mark (?) indicates where an accurate categorization cannot be made based on current published work and further research is needed. In the case of the Horsfield's bronze cuckoo, learning does not appear to employ auditory input of a model sound and as such, the other categories within that dimension are labelled not applicable (N/A). All classifications are made based on current available knowledge (see reference numbers within circles for evidence used for classifications), and should be updated as new information comes to light.

Starting from the aforementioned definition, we consider vocal production learning to be an ‘umbrella’ term covering a number of behavioural dimensions, many of which appear to be continuous, while others are discrete (figure 1). Below, we break down vocal production learning into dimensions to produce a more fine-grained view of this complex behaviour and to show how these dimensions can alter vocal outputs in different ways. In doing so, our aim is to produce a behaviour-focused framework within which it would be possible to categorize the varied vocal production learning abilities of different animals, facilitate inter-species comparisons of these behaviours and ultimately, elucidate the underlying mechanisms.

### (a) MODEL: vocalizations converge on or diverge from the model

The examples of vocal production learning given above result in learning to make vocalizations more similar to an auditory model. However, vocal production learning can also involve modification of vocal outputs so that they become different from the auditory model. Diverging from the model can be beneficial if the vocal learning serves to produce a signal necessary for individual recognition that should be species typical, yet distinct from others that they encounter [2,3]. Bottlenose dolphins use vocal learning in the development of individually distinctive signature whistles seemingly by modifying whistles that they have heard in their environment [92]. Rapid call convergence and divergence has been observed in playback experiments with several parrot species. The degree and direction of the resulting changes seems to depend on the social dynamics of the species and individuals concerned [93]. To diverge from a model, a template based on the model would still form, but the vocal outputs would be modified to avoid similarity to the template. It is possible that a second auditory template could form if reliable production of the divergent vocalization is needed. In practice, it may be difficult to determine whether an observed divergent vocalization is the result of active modification to differ from a model, or matching to another model that is unknown to the observer. While convergence on a model is the most widely (and easily) observed behaviour, further work is needed to determine the spread of, and mechanisms underlying, divergence from a model.

### (b) MODEL: accuracy of the copy

The fidelity of a copy produced during vocal production learning has previously been well laid out in relation to song learning in birds by Beecher & Brenowitz [6]. They describe a continuum from high fidelity (faithful imitation), to medium fidelity (improvisation that introduces variation to sounds copied from a tutor), to low fidelity (invention that is species typical but does not resemble a tutor, and may not even require a tutor). In this latter case, the bird may still require exposure to songs in order to produce a species-typical song. For instance, grasshopper sparrows (*Ammodramus savannarum*) must have heard normal songs in order to develop normal, species-typical warble songs instead of impoverished songs with abnormal warble notes [94]. Nevertheless, the songs resulting from exposure to a tutor do not resemble the tutor song more than they resemble another normal grasshopper sparrow song. So, there is an effect of experience, but it does not translate into copying. In a species that produces excellent copies of model sounds heard earlier a clear template must have been formed. In a species requiring exposure to song, but not resulting in copying, the exposure may be needed to stimulate the development of a latent song programme or to learn the appropriate characteristics of species-typical songs. These different outcomes of exposure suggest different underlying processes by which experience can influence vocal development and these contrasting phenomena need further exploration.

There may be circumstances where copying fidelity is of more or less importance during vocal production learning. For example, learning that results in convergence towards a model (see the previous section) may require medium to high fidelity because accuracy brings benefits to the copier. An adaptive need to diverge from a model may employ precise avoidance of the model, or could employ low fidelity copying in combination with improvization or innovation. For example, bottlenose dolphins address each other by copying their individually distinctive signature whistles [95,96]. While the overall modulation pattern of model whistles is produced accurately in these copies, multi-dimensional scaling of specific acoustic parameters of copies often result in substantial differences from the whistle that is being copied, suggesting a possible use of such mechanisms to make copies recognizable as such to eavesdroppers [97]. In some species of songbird, the ability to accurately imitate song elements or sequences of song elements may be targets of inter- and intra-sexual selection because it could signal aspects of individual quality and fitness [98–101], including motivation and/or ability to attend to social cues, use social information and form social bonds [102,103].

Importantly, when assessing the precision of the match and the importance of fidelity, we must strive to consider this from the animal's perspective. Although we may be able to quantify subtle differences on a spectrogram, differences in the fidelity of a copy are only relevant when and if the study species can perceive a difference. Conversely, using our own assessment of a ‘good’ match may lead to overlooking important features that are relevant for the study species.

### (c) DEGREE: type of vocal modification

Production learning may involve modifications of existing vocalizations or the production of completely novel vocalizations. In addition, vocal outputs can be modified by learning individual calls or learning sequences of calls (see §5 for further discussion of this issue). In all these cases, a key challenge is to determine what constitutes a novel sound pattern. In determining whether the vocalization observed is a novel sound, or a modification of an existing sound, the complete vocal repertoire for an individual is needed. While this requires knowledge of an individual's repertoire before it has been exposed to the learning experience, this knowledge can seldom be claimed to be complete because it is very difficult to be sure that all rarely used vocalizations or heavily context-specific vocalizations have been captured. However, in practice, larger qualitative or quantitative changes in vocal structure, in particular, when they are towards some identifiable model, will be strong indicators of learning. In the cases of animals imitating human speech or a songbird copying a mechanical sound, it is obvious that a novel vocalization is being produced. It is not yet known whether modification of existing calls and production of novel calls employ the same or different neural mechanisms [5,17], but this is a key area for future research, as understanding the mechanisms will be highly beneficial not only to better categorize the behaviour, but to understand how the behaviour arises.

Modification of calls can employ different physical mechanisms operating on the airflow, the sound-producing organ or the filtering vocal tract that can co-occur or to some extent independently affect a call's spectral structure. While duration and amplitude need control of the respiratory system this needs to be coordinated with the configuration of the sound source. Similarly, frequency modulation patterns are most prominently influenced by the phonatory system [2] but increasing amplitude by controlling respiratory pressure can lead to an increase in frequency parameters and occurrences of nonlinear phenomena, again affecting the spectral structure. Another important way of learning to modify sounds is by changing the way in which the upper vocal tract filters or emphasizes parts of the signal produced by the sound source (e.g. the vocal folds in most mammals or syrinx in birds) [104]. Janik & Slater [2] called this filter learning and highlighted its possible involvement in human speech owing to the importance of these structures to encode contrasts in vowels and consonants [105].

### (d) DEGREE: breadth of learning

Vocal learners differ greatly in the breadth or narrowness of the range of vocalizations they can learn to produce. Several songbird species are strongly biased towards learning to produce only species-typical vocalizations [72]. Some animal species can mimic species-atypical sounds such as human speech (e.g. parrots [74], elephants [34], seals [77]) and some bird species are able to mimic a huge range of sounds produced by different animals (e.g. mynah birds, starlings, drongos [106,83]), or even non-biological sources in their environment. For example, lyrebirds can copy the sounds of a range of other animals or even a chainsaw or camera shutter from their environment [107,108]. Beecher & Brenowitz [6] refer to this variation in breadth of learning as the degree of canalization.

The breadth or narrowness of the vocalizations an animal can learn to produce may result from different selection pressures leading to a more or less narrow choice of models and vocalizations. Morphology may also preclude species from producing certain types of sounds because of the limitations of their vocalizing organ. Neural constraints may determine whether or not the animal recognizes sounds as a relevant model to attempt to copy and whether motor programmes are flexible enough to copy atypical models.

Environmental factors or motivation may also be key influences in determining the breadth of learning for many species. The social relationship between tutor and tutee and the presentation mode (live or playback) may affect the motivation to copy vocalizations [102]. Similarly, under normal circumstances, and in the normal environment of the animal, it may be the case that only species-typical sounds are learned. However, under unusual conditions (e.g. captive rearing, atypical social bonding with heterospecifics), animals may mimic unusual sounds. Such a situation is exemplified by the individual cases of Hoover the harbour seal (*Phoca vitulina*) [109], Koshik the Indian elephant (*Elephas maximus*) [34] and most recently by a case of Ripper, an Australian musk duck (*Biziura lobata*) [110], which were each observed to mimic human speech-like sounds. Seals, elephants and musk ducks are not routinely observed to mimic human speech, but in each of these cases, the individuals were reared in isolation from conspecifics and formed strong bonds with human keepers. It is hypothesized that in the absence of exposure to conspecific calls and because of the strong human social bonds formed, the animals were motivated to mimic human sounds that would not normally be considered a relevant model by these species. The age at which bonding occurred might also strongly influence whether a model is considered relevant (see also the section on timing below). In any case, these examples show us that the necessary peripheral morphology and the neural circuitry is present in seals, elephants and musk ducks, but that they will only mimic human speech sounds under extreme circumstances. As such, we must be careful to consider what an animal is ‘capable’ of doing if there is only an absence of evidence regarding the breadth of their vocal production learning capacity.

### (e) TIMING: when learning takes place

The age or seasonal context during which production learning can occur differs between species. In some birds, there is evidence that the formation of a template used for vocal learning may already start before they emerge from the egg. In the superb (*Malurus cyaneus*) and red-backed (*Malurus melanocephalus*) fairywrens, females give a specific call when incubating their eggs and chicks appear to incorporate these into their begging calls, suggesting that the calls are memorized *in ovo* [111–113]. For several songbirds, the phase for song learning is well defined. For example, zebra finches and white-crowned sparrows (*Zonotrichia leucophrys*) form their song memory (template) predominantly during a juvenile sensitive phase. They progressively match their later vocal output to this template and when the songs are crystallized and stereotyped the birds are then no longer able to learn new songs [114,115]. Other species, such as parrots are able to continue to learn new vocalization as adults, and some birds such as canaries or starlings go through a sensitive phase seasonally, learning new sounds or songs only during a specified annual window (e.g. [116,117]).

In mammals, the period during which vocal learning can take place has rarely been studied. Humans are able to learn novel sounds throughout their lifetime, but there appears to be a sensitive period for learning to distinguish or reproduce all aspects of the speech sounds of a language [118]. Humpback whales and bottlenose dolphins are clearly capable of learning novel sounds throughout their lifetimes [119]. Single case reports, such as those for Hoover the seal [109] or Koshik the elephant [34], suggest that their production of learned vocalizations co-occurred with sexual maturation stages, but it appears likely in these cases that the learning of the auditory templates took place earlier in life as juveniles. Whether as mature adults they continued to add novel vocalizations is unknown. In a controlled experimental study, three grey seals less than a year of age were able to both learn and produce speech sounds while still juveniles [77]. For most non-human mammals, controlled experiments or long-term monitoring have not been performed and these are crucial to allow the determination of sensitive periods for vocal production learning.

### (f) TIMING: length of time learning takes

Modification of existing sounds can involve a rapid ‘step-change’ or gradual changes over time to produce the desired vocalization. Both of these can ultimately result in novel call types, but one happens immediately, the other, gradually. Memorizing a template and shaping vocal output accordingly may each take time, but a template may also be memorized rapidly in some species with initial vocal output already matching the model. For example, dolphins can be trained to imitate novel artificial sounds after the first exposure to a synthetic playback sound [40], and a nightingale (*Luscinia megarhynchos*) may reproduce a series of different songs it has heard only 20 times [120]. Converging on calls may also vary in the amount of exposure (trials) required. Some parrot species, such as the orange-fronted conures (*Aratinga canicularis*) can converge their contact calls towards a playback of a different contact call during a single playback session [87]. Conversely, in pale spear-nosed bats, many trials over approximately 30 days were required to learn to produce a small pitch shift [121].

### (g) TIMING: how long the learning is retained

In the previous section, we discussed whether animals rapidly learn to produce a new call type, or gradually learn to modify their call to approach the properties of the template. Related to this dimension is also the question of whether the internal auditory template that the animal is aiming to produce is stored in long term or only short-term memory. It is clear that in many classical cases of vocal production learning, such as songbird song learning, the internal auditory templates are maintained in long-term memory. For many songbirds, it has been shown that the learning process consists of two phases, a first phase of sensory learning, in which an auditory model is memorized as a template, and a second phase of sensory-motor learning in which the template guides the developing vocal outputs [90]. The songbird system thus puts strong emphasis on the long-term formation and use of a template.

Formation of a transient template in short-term memory would only allow for immediate matching of acoustic parameters or require repeated auditory input. If learning involves rapid production of new call types, such as in parrots and dolphins it is not always clear whether these templates are retained in long-term memory unless the resulting vocalizations are produced long term. Dolphins not only learn to imitate sounds immediately, but can be trained to use earlier mimicked sounds as object labels [40], indicating long-term storage of internal templates for these novel produced sounds. In the case of Alex, the African grey parrot, it seems likely that the internal templates for the words he learnt were stored long-term as these sounds continued to be produced over a long period [74]. Tests of his comprehension and use of labels included objects that he might only see once per week [122], but he still could name them, so the internal templates were likely to have been retained at least this long. Differentiating between short- and long-term retention of learned templates will require experiments specifically designed to address this. For example, if matching to a sound that had been matched in the past is faster than the matching of a new sound, it would imply that some knowledge of the first template was retained in long-term memory. Exploring examples where short-term versus long-term retention of templates are observed could illuminate the mechanisms underlying these types of vocal production learning.

## 5. Grey areas in classification of vocal learning

Although we have endeavoured to produce a clear typology of vocal learning, some grey areas of classification remain. In some cases, it is not apparent if or when a learning process lies behind the observable behaviour. In other cases, the mechanisms behind behaviours identified as vocal variation, usage learning and/or production learning may be hard to distinguish between, or may even be co-occurring. In this section, we will discuss some of the outstanding ‘grey areas’ that hamper clear categorization of vocal learning across species.

In this paper, we have retained a reasonably strict definition that vocal production learning requires learning from an auditory model, and the formation of a template to which the vocal output is compared (see §4). However broader definitions of vocal production learning can be used in which the requirement for hearing an auditory model during the learning process is omitted [85]. An example of learning under this broader definition may be in Horsfield's bronze cuckoo (*Chalcites basalis*). The offspring of this brood parasitic species are reared by a foster species and after a few days produce begging calls that are quite similar to those of host young even though no host young are present. Experiments showed that this is the result of the gradual changing of a ‘standard’ begging call so that it that becomes similar to host begging calls over a short period [123]. As there are no offspring of the foster species in the nest, there is no auditory model available to guide the change. The mechanism underlying the vocal change is suggested to be a type of operant learning [85]: parents are likely to provide more food when the chick begging call resembles what parents expect to hear from their offspring [123]. If the cuckoo receives limited responses to its begging, it starts to produce more variable calls, which might be considered improvizations on the cuckoo begging call. Those variants resulting in more food being delivered become more common and may be further varied so that it reaches a version which results in stable parental feeding. In this case, one might say that if there is a template, it is not in the mind of the learner, but in the mind of the rewarder [85]. Reward-driven changes in vocalizations have also been shown in systematic experiments on budgerigars [124], cowbirds [125], bats [33] and pinnipeds [126]. If the reward-driven changes are rather limited it is difficult to rule out that they were a consequence of usage learning, resulting in the production of a previously existing (but possibly rare) variant of a call. Conclusions on the production mechanism of apparently novel or idiosyncratic sounds are especially hard to draw where comparisons to the natural repertoire and its inherent flexibility in wild conspecifics are lacking. If, however, reward-driven changes result in vocalizations well outside the known range of a species, such reward-driven vocal production learning may employ different mechanisms from those that rely on exposure to and subsequent matching of an auditory model by the learner.

Animals and humans may use a variety of learning mechanisms to learn to produce new sequences of vocalizations. In animal communication, learning sequences may involve simple linear arrangements of a small number of vocalizations (e.g. in primates [127–129]), or more complex arrangements such as songs that can involve combining calls or song syllables into repeating subunits of phrases or themes, some of which display complex syntax (e.g. birds [130–132], whales [133]). One example is Bengalese finch (*Lonchura striata* var. *domestica*) songs, which consist of syllable sequences that can be arranged in various orders, the sequence of which is affected by experience [134]. In some animal species, the rearrangement of vocalizations alters the functional response, which has been suggested to represent a simple combinatorial syntax (e.g. pied babblers (*Turdoides bicolor*) [135]). If an animal is reinforced to produce two vocalizations in a specific order, then associative learning can lead to sequence learning, and this would be categorized as usage learning. Sequence learning plays an important role in vocal production learning in some species as well. If learning a novel sequence of sounds leads to co-articulatory effects that modify the vocal output to produce a new sound [136], this could then be a case where both vocal usage and production learning could be argued. In humans and birds, there is evidence that the mechanism by which sequencing is learned is distinct from that which matches the spectral element structure to a template. In humans, learning the acoustic structure of phonemes appears to occur via a separate process to their sequencing in speech [137]. When birds learn a new song type, they have to acquire not only the elements that they need to produce but also the sequence and timing of these. There is also evidence that these two components are learned separately in songbirds, but that the processes are tightly integrated, similar to the processes that occur during human speech acquisition [134,138–140]. Currently, it is unclear whether this type of sequence learning uses the same neural structures as when the bird learns to give a call in a new social context or whether it shares components used in vocal production learning. Sequence learning is a key area for future study to understand processes by which it can take place and how they relate to, or overlap with the features and mechanisms underlying other aspects of vocal learning behaviour.

## 6. Mechanisms

The most accurate and useful classification system of vocal learning is one in which the behavioural categories are mirrored by mechanistic boundaries. Mechanistic understanding is thus key to resolving at least some of the grey areas in classification, such as those discussed above. For these reasons we discuss known and possible mechanisms underlying vocal learning, and in box 1 outline some key outstanding questions for future research that will lead to an improved typology and understanding of vocal learning.

Studies in songbirds and humans demonstrate that vocal learning exploits specialized circuitry, but it is not yet known to what extent mechanisms underlying modification of existing vocalizations and learning novel sound types are overlapping or discrete in the brain, or how they evolved. Tyack posits that fine tuning of the acoustic features of existing calls in a repertoire represents a limited form of vocal learning, while the acquisition of a completely novel call type represents complex vocal learning, and that these are distinct behaviours involving different brain circuitry, such that complex vocal learning may have been achieved by supplementing subcortical circuits with new cortical connectivity [5,17]. Petkov & Jarvis [4] suggest that these extremes represent minima and maxima of a stepwise continuum and that existing circuits have been gradually built upon to increase behavioural complexity [4,141]. A major challenge for future research will be to elucidate the neural mechanisms underlying the different aspects of vocal modifications and vocal learning.

### (a) Vocal usage learning

In this framework, usage learning covers a range of behaviours (associating existing sounds with novel contexts, learning new sequences of existing elements or altering the timing of sound production) that may employ different mechanisms. Furthermore, it is currently unclear how distinct or overlapping neural circuits involved in vocal usage learning are with those needed for vocal production learning. Vocal usage learning is widespread, while most usage learners seem to lack the capacity for vocal production learning, suggesting specialized circuity. However, this does not mean that there is no overlap in circuitry between the components of these behaviours. For example, in the song control system of zebra finches the premotor nucleus RA (nucleus robustus arcopallialis) and the projections that it receives from another telencephalic nucleus, HVC (proper name), are involved in the production and timing of non-learned calls in signal exchanges [63,142], showing that usage and production learning can share some neural structures. Thus, it may be that there is some shared recruitment of brain regions or circuits to subserve vocal usage and production learning, but that production learning requires the development of further specialized circuitry, particularly because usage learning can occur without the need for auditory input or matching of an auditory template. Instead, it can occur via ‘learning from success'—i.e. learning that using a specific call is more likely to achieve the desired response [5,8,123,125,143]. This makes it likely that different pathways are involved in error-correction in vocal usage and vocal production learning.

### (b) Vocal production learning

Vocal production learning requires exposure to an auditory model, formation of a memory of the target sound (template), control over anatomical structures to produce an approximation of the desired sound, and auditory feedback to perceive and adjust vocal output as needed. Neural circuits that control the perception, memorization, template matching and motor production of learned vocalizations crucial for vocal learning are likely to have been built upon existing circuits over evolution. Although neural contributions to this complex trait must necessarily involve distributed circuitry throughout the brain, a few key pathways currently stand out as being specialized in vocal production learners [104,141].

Songbirds provide the best mechanistic understanding of the neural mechanisms involved in vocal production learning, in particular how model sounds are learned to form an auditory template and how auditory feedback during attempts to copy the sound allows the bird to converge on a model. In birds, the caudomedial nidopallium (NCM) has been proposed as a candidate site where tutor song memories are stored (reviewed in [144]). The NCM is an auditory region in the songbird ascending auditory pathway that, along with other forebrain and pallial nuclei, are viewed as analogous to the mammalian auditory cortex [145]. The NCM and another forebrain auditory region, the caudomedial mesopallium (CMM) are specialized for auditory learning and memory of conspecific vocalizations [146–150]. In an avian vocal mimic (budgerigar), vocalizations by conspecifics activate the NCM and CMM even when the sounds made by the conspecifics are unusual (e.g. a conspecific mimicking human speech [151]). In particular, data showing that activation of NCM predicts the accuracy with which zebra finch tutor songs are imitated [152–156] suggest that the precision of song imitation depends greatly upon the fidelity of memory encoding and consolidation of tutor song in this region. However, it has also been suggested that memories used to guide the vocal production of a learned auditory template are separately stored from those used to perceive and remember other conspecifics [157]. Instead of being stored as an auditory template in NCM, auditory templates that the bird is trying to produce are mapped on to and encoded in vocal-motor circuits (see [157–159]). In line with the idea that NCM is not involved in song imitation, lesions to NCM in juvenile zebra finches before or after tutor song exposure did not prevent them from learning to imitate tutor songs [160,161]. Juvenile zebra finches could also be optogenetically ‘tutored’ to sing experimentally specified song element durations by stimulating activity of premotor nucleus HVC via an upstream auditory region (NIf), i.e. independent of NCM. This effect occurs even if they have the opportunity to learn from a live adult male tutor [162]. Discovering whether tutor song is also stored in motor circuits of female songbirds that do not sing may provide further clarification on this matter.

Auditory feedback is a necessary element for shaping vocalizations in vocal production learning. Conceptually, auditory feedback of one's own vocalizations can be compared to a memorized auditory template (comparator-type models) or by mapping auditory memories of tutor song onto vocal-motor commands so that as the bird sings, the motor connections that produce the desired auditory feedback are strengthened (inverse-type models, e.g. [163], see [158,164] for overviews of inverse models in songbirds). Nonetheless, extensive connectivity between motor and auditory areas is necessary for neuronal integration of auditory–vocal information. In humans, a dorsal auditory–parietal–motor cortical circuit has much stronger connectivity via the arcuate fasciculus compared to non-human primates [165]. Vocal learning birds also show novel connectivity between auditory and vocal learning nuclei [141], as well as connectivity between song motor regions and ventral tegmental area dopaminergic neurons that provide reinforcement signals contingent on auditory feedback to guide learning [166–169]. Such auditory–vocal integration circuits may link the perception of a model sound to the programming of a novel motor output and/or matching of the produced sound to the model.

In vocal learning birds and humans, a direct forebrain-motor control pathway has been identified that is thought to enhance control over the syrinx (birds) or larynx (humans) and facilitate learned vocalizations (but see also comments on dolphins in the peripheral mechanisms section, below). The laryngeal motor cortex is a specialized region of the ventral primary motor cortex that connects to primary motor neurons in the brainstem that in turn innervate laryngeal muscles [170,171]. Species such as cats, which are thought not to be vocal production learners [172], innervate these muscles via indirect connections between the motor cortex and primary motor neurons [173]. By contrast, direct, strong mono-synaptic connections have been identified in oscine songbirds that learn their songs and in humans, and it has been hypothesized that these are required for vocal production learning [174]. Testing this hypothesis more broadly requires studying a range of species with varying levels of vocal production learning. These auditory and vocal-motor connections are poorly studied in most other vocal learning species—particularly in vocal learning mammals. This is in part owing to the inaccessibility of many mammalian vocal learners, but also the practical challenges that come with performing tracing or magnetic resonance imaging based studies in large and/or aquatic animals. Thus, determining the presence or absence of these connections in other more tractable vocal learning mammals such as bats or seals, coupled to behavioural studies of vocal learning capabilities, will be important to test these hypotheses and determine the necessary neural prerequisites for the range of vocal learning abilities found across species.

### (c) Peripheral mechanisms

The ability to vocally match a memorized model is not only constrained by the fidelity of memories or the learning circuitry, but also by the capabilities of the vocal production apparatus. The production of complex and varied vocalizations in birds and mammals employs sound source (e.g. larynx or syrinx) and sound filtering (e.g. orofacial control with lips, tongue, nasal passages, jaws and/or beaks) [104]. The structure of the larynx is highly conserved across mammals, so peripheral constraints of this vocal apparatus are expected to be less important for determining the breadth of vocal production learning within mammals than are neural constraints. For example, non-human primates have the physiological capacity to produce many human speech-like sounds [175]. Yet in practice, this does not occur even in the context of intensive training attempts, which is hypothesized to be owing to a lack of the appropriate learning circuitry [174]. However, the larynx is not always employed in mammalian vocal production learning. Dolphins are among the best mammalian production learners but do not use the larynx to produce sound. Sound originates in a newly evolved structure in the upper nasal passages called the dorsal bursae complex that includes two pairs of phonic lips that can produce two sounds independently at the same time [176,177].

The avian equivalent of the larynx is the syrinx. Bird groups show more differences in the structure of their syrinx and the number of muscles that can modify its shape, than is observed for the mammalian larynx. So, it is conceivable that owing to this, different bird species have greater differences in the range of possible sounds they can produce. As an example: some species have two sound sources, one in each primary bronchus [178,179]. This latter structure allows some species to make two different types of sounds at the same time (e.g. brown-headed cowbird, *Molothrus ater ater*), which cannot be done with just one source [180,181]. It is, therefore, important to consider the peripheral morphological properties of a species to understand the potential constraints on vocal learning abilities.

## 7. Outlook

In this paper, we have aimed to build on the strong foundations of Janik & Slater [2,3], Petkov & Jarvis [4], Tyack [5] and others (e.g. [6–10]) to define a framework for understanding vocal learning across species. We contrasted non-learned vocal variation with vocal usage and vocal production learning. We divided vocal production learning into dimensions to display the complexity of this behaviour and allow a more fine-grained understanding of the components of vocal learning. This framework illustrates clear differences in behaviour, even between the most widely cited examples of vocal production learning species (figure 1). For example, while both a parrot and a zebra finch are capable of performing vocal production learning, zebra finches require an extended sensory-motor learning phase, while parrots quickly mimic a new auditory model. Furthermore, while zebra finches copy a narrow set of sounds during a limited developmental period, parrots can learn a much greater range of sounds, and continue to do so throughout life. Thus, while both are avian vocal learners, there are striking differences in their learning. Extending such a classification system to all vocal learning species would allow informed selection of species in which to best explore the mechanisms underlying a specific aspect of the set of behaviours that make up the dimensions of vocal learning. Similarly, extending this classification system would show which dimensions are most common or most rare, and which frequently co-occur, which may shed light on mechanistic or evolutionary relationships between dimensions.

A typology is only as good as the data on which it is based, and it is clear that vocal learning suffers from a severe absence of evidence in most species studied, as well as outstanding ‘grey areas’ where classifications cannot be clearly made, as is evident from the question marks (?) displayed in figure 1. Indeed, outside of songbirds (or even among them) we should consider the data to be largely incomplete. Thus, we acknowledge that the classifications outlined herein are based on the current available evidence. For this reason, figure 1 is not meant to be exhaustive, but is meant to represent a classification of a small selection of species based on currently available knowledge. Through much needed naturalistic and experimental observations, future studies are likely to reveal a wider range of species with vocal learning abilities than currently identified. But we hope that, as new evidence comes to light, our framework will make it possible to align these species with what we know now. It would also be useful (although challenging) if future studies were able to reveal which species lack certain abilities. In the meantime, we must be clear about the difference between what an animal is *capable* of doing versus what we have been able to *observe* it doing. This is perfectly exemplified by the cases of Hoover the seal and most recently Ripper the Australian musk duck [109,110]. These single animal observations were so striking that they were able to indicate vocal production learning capacity for a species; however, they might easily have gone undiscovered without the right conditions fortuitously arising and being reported upon.

Our framework classifies the behavioural dimensions of vocal learning, in order to select species for the study of mechanisms of learning, but this categorization is by necessity an approximation. The ‘true’ definitions will match the underlying mechanisms, which may not align perfectly with the current behavioural distinctions. As such, determining the underlying neural mechanisms and demonstrating which behavioural distinctions are well supported by such mechanistic evidence is ultimately needed. Understanding mechanisms will help us resolve at least some of the grey areas of classification and determine the most important behavioural features on which to focus. For example, it might help resolve what is a ‘novel’ call, or whether a small change in vocalization employs the same process as learning to produce a very large change. It could illustrate if an animal learning a species-typical call type is performing the same task as one learning to mimic human speech, or if these are actually distinct processes. It is crucial that such work be performed comparatively in various avian and mammalian species to determine if the observed mechanisms are a universal hallmark of the behaviour, or a feature of the clade or species under study [7]. Exploring the underlying mechanisms will also reveal how the behaviours have evolved—e.g. via building on existing brain circuitry in a continuous fashion to improve skills as opposed to a dramatic step-change to introduce a new ability. Currently, mechanisms have not been sufficiently explored in enough species to make these distinctions or to generalize on how mechanisms align with behaviour. As such this is a clear area where future research is needed (see Box 1). We hope that the framework outlined here will aid in the long-term goal of producing of an integrated, mechanistic understanding of vocal learning across species and shed light on the evolution of vocal learning, including that in humans.

## Data accessibility

This article has no additional data.
